# CD154 Blockade Alters Innate Immune Cell Recruitment and Programs Alloreactive CD8^+^ T Cells into KLRG-1^high^ Short-Lived Effector T Cells

**DOI:** 10.1371/journal.pone.0040559

**Published:** 2012-07-05

**Authors:** Ivana R. Ferrer, Maylene E. Wagener, Mingqing Song, Mandy L. Ford

**Affiliations:** Emory Transplant Center and Department of Surgery, Emory University, Atlanta, Georgia, United States of America; University of California, San Francisco, United States of America

## Abstract

CD154/CD40 blockade combined with donor specific transfusion remains one of the most effective therapies in prolonging allograft survival. Despite this, the mechanisms by which these pathways synergize to prevent rejection are not completely understood. Utilizing a BALB/c (H2-K^d^) to B6 (H2-K^b^) fully allogeneic skin transplant model system, we performed a detailed longitudinal analysis of the kinetics and magnitude of CD8^+^ T cell expansion and differentiation in the presence of CD154/CD40 pathway blockade. Results demonstrated that treatment with anti-CD154 vs. DST had distinct and opposing effects on activated CD44^high^ CD62L^low^ CD8^+^ T cells in skin graft recipients. Specifically, CD154 blockade delayed alloreactive CD8^+^ T cell responses, while DST accelerated them. DST inhibited the differentiation of alloreactive CD8^+^ T cells into multi-cytokine producing effectors, while CD40/CD154 blockade led to the diminution of the KLRG-1^low^ long-lived memory precursor population compared with either untreated or DST treated animals. Moreover, only CD154 blockade effectively inhibited CXCL1 expression and neutrophil recruitment into the graft. When combined, anti-CD154 and DST acted synergistically to profoundly diminish the absolute number of IFN-γ producing alloreactive CD8^+^ T cells, and intra-graft expression of inflammatory chemokines. These findings demonstrate that the previously described ability of anti-CD154 and DST to result in alloreactive T cell deletion involves both delayed kinetics of T cell expansion and differentiation and inhibited development of KLRG-1^low^ memory precursor cells.

## Introduction

Current immunosuppressive regimens in organ transplantation require life-long administration and result in off-target toxicities such as nephrotoxicity and cardiovascular and metabolic complications [1]. Considering these significant co-morbidities, much work over the years has focused on the development of novel modes of immunosuppression. The development of costimulation blocking molecules has been the basis for research by several groups to specifically target and inhibit the full activation of alloantigen-specific T cells at the time of transplantation. One of the most effective pathways for therapeutic intervention is the CD154/CD40 pathway, blockade of which results in profound inhibition of graft rejection and in some models the induction of transplantation tolerance [2]–[4]. However, translation of therapeutic blockade of this pathway has been stymied by the observation of thromboembolic complications in pilot clinical trials as a result of the expression of CD154 on platelets [5]. Nevertheless, understanding the altered differentiation programs initiated in alloreactive T cell populations under conditions of CD154 blockade remains an important goal in the ongoing pursuit to harness the therapeutic potential of this pathway.

In order to study the effects of CD40/CD154 pathway blockade on donor-reactive T cell responses to a transplant, we employed an allogeneic skin graft (SG) model in which anti-CD154 monoclonal antibodies (mAb) were administered in combination with donor specific transfusion (DST) as previously described [4], [6], [7]. DST provides a large bolus of antigen presented by relatively inert APCs [8], stimulating antigen-specific T cell activation by providing “signal one.” Other groups have also demonstrated the potent effects of combined DST and costimulation blockade in the prolongation of islet, cardiac, skin and kidney allograft survival in murine and nonhuman primate models [4], [6]–[11]. Although it has been generally accepted that CD154 costimulation blockade leads to anergy [12] or deletion [13], [14] of recently activated T cells, the mechanism by which DST and anti-CD154 mAb synergize to induce these effects on the alloreactive T cell population remains incompletely understood.

In order to assess the differential impact of DST and anti-CD154 mAb on the programming of donor-reactive CD8^+^ T cell expansion, contraction, and differentiation over time, we performed longitudinal analyses on the donor-reactive CD8^+^ T cell responses. We hypothesized that the previously observed deletion of graft-reactive CD8^+^ T cells following anti-CD154/DST treatment was the result of differential programming of these cells following encounter with alloantigen [8], [12]. Recently, studies of viral-specific CD8^+^ T cell responses have revealed programmed differentiation of antigen-specific T cells into either long-lived memory precursors or short-lived effectors as early as four days post-infection [15]. These differentially programmed cells can be segregated on the basis of their expression of KLRG-1 (killer cell lectin-like receptor G-1), in that KLRG-1^high^ cells represent short-lived effectors, while KLRG-1^low^ antigen-specific CD8^+^ T cells distinguish the long-lived memory precursors [15], [16]. As compared to KLRG-1^low^ cells, KLRG-1^high^ cells go on to express lower levels of Bcl-2, CD27, and CD62L, and higher levels of GzmB. Functionally, KLRG-1^high^ cells are compromised in their ability to produce IL-2, an important T cell autocrine growth factor [15], [16]. Finally, adoptive transfer recipients of KLRG-1^high^ cells have been shown to have poorer recall potential upon secondary rechallenge as compared to those receiving KLRG-1^low^ cells, consistent with diminished ability to survive and differentiate into long-lived memory cells [15]. Here, we assessed the impact of anti-CD154 and DST to induce distinct differentiation programs in graft-reactive CD8^+^ T cell responses. Specifically, anti-CD154 treatment functioned to reduce the magnitude of the alloreactive T cell response by delaying CD8^+^ T cell expansion and increasing the proportion of KLRG-1^high^ short-lived effector cells. In contrast, DST treatment prevented alloreactive CD8^+^ T cells differentiation into multi-cytokine producing effectors.

In addition to its potent effects on adaptive immune responses, CD154 can play a major role in the activation of innate immunity. For example, in the setting of tissue injury and inflammation, CD154-expressing platelets are activated and have been shown to induce upregulation of adhesion molecules on and chemokine secretion by endothelial cells in a CD40-dependent manner [17]. Because chemokines that recruit innate immune cells are expressed early in the wound healing process of skin grafts [18], we aimed to determine whether blockade of CD154 also influences the chemotactic signals delivered to graft-infiltrating leukocytes. In this study, we observed that animals treated with CD154 blockade had significantly impaired expression of CXCL1/KC, CCL3/MIP-1α (macrophage inflammatory protein-1α), and CCL5/RANTES (regulated upon activation normal T cell expressed and secreted) in the allografts compared to untreated controls. Thus, blockade of CD154 impacts both innate and adaptive immunity to prolong allograft survival.

## Materials and Methods

### Ethics Statement

This research was approved by the Emory University Institutional Animal Care and Use Committee. All animals were treated ethically according to Emory University IACUC protocol 2001175.

### Mice

B6-Ly5.2/Cr (H2-K^b^, CD45.1) and BALB/c (H2-K^d^, CD45.2) mice were obtained from the National Cancer Institute (Charles River, Frederick, MD).

### Skin Transplantation, Donor Specific Transfusion and Antibody Treatment

Full thickness BALB/c tail and ear skins were transplanted onto dorsal thorax of recipient mice and secured with adhesive bandages for 6 days. DST was the adoptive transfer of whole splenocytes, given as a single dose of 10^7^ splenocytes i.v. on the day of transplantation. Anti-CD154 treatment (MR1, BioExpress, West Lebanon, NJ) was administered i.p. at 500μg/ dose on the day of transplantation as well as on days 2, 4, 6 post-transplantation, where indicated. Skin grafts were monitored over time and declared rejected when <10% viable graft remained.

### Activated T Cell Surface Staining and TruCount Analysis

At indicated time points, splenocytes were removed and disrupted with glass slides. Cells were stained with antibodies against CD4 and CD8 (Invitrogen), CD62L (BD Pharmingen), CD44 (eBiosciences), and KLRG-1 PE (Southern Biotech). Absolute counts were obtained by using TruCount tubes (BD Pharmingen). Samples were run on a LSR II Flow Cytometer (BD Pharmingen). Data were analyzed using FlowJo software (Treestar, San Carlos, CA).

### Histology and IHC Quantification

Skin grafts were removed and placed in cryomolds with OCT Embedding Compound (Tissue-Tek, Hatfield, PA) and frozen on dry ice on day 7 post transplantation. Longitudinal sections of skin grafts were cut 5 μm thick with a cryostat (Leica CM 1850, Leica Microsystems, Wetzlar, Germany) and mounted on Superfrost Plus microscope slides. Slides were fixed with 100% acetone and used for H&E as well as immunohistochemical staining. CD8a (BD Biosciences) and Neutrophil Marker, Ly6b, (Santa Cruz Biotechnology, Santa Cruz, CA) antibodies were used for CD8^+^ T cell and neutrophil immunohistochemical detection, respectively, by 3,3 diaminobenzidine (DAB) peroxidation and counterstained with haematoxylin. An Olympus BX43 microscope was used for visualization.

The whole slide digital images were captured using the Aperio ScanScope XT Slide Scanner (Aperio Technologies, Vista, CA) system with a 20× objective. Images were viewed and analyzed with ScanScope software using positive pixel count algorithm (Aperio). Several fields (8–10) of epidermis and dermis areas were measured in each section. The ratio of total strong positive vs. area measured was used as the quantifying parameter.

### RNA Isolation and Chemokine RT-PCR

Mice were sacrificed and skin grafts were extracted and placed in RNA*later* (Qiagen) at 4°C until ready for use. RNA was isolated from skins using RNeasy Fibrous Tissue kit (Qiagen), according to manufacturer's instructions. Reverse transcription of RNA into cDNA was performed using TaqMan reverse transcription kit (Roche). CXCL1, CCL3, CCL5 real-time PCR assays (Applied Biosystems) were run on a 7900HT Real-Time PCR System (ABI).

### T Cell Intracellular Cytokine Staining

To measure IFN-γ (eBiosciences) and TNF (BD Pharmingen) production by donor reactive cells, single cell suspensions of responder splenocytes from transplanted mice were stimulated with BALB/c splenocytes in the presence of 10μg/mL Brefeldin A for 4 hours *ex vivo*. An intracellular staining kit (BD Biosciences) was used according to manufacturer's instructions. Samples were run on a LSR II Flow Cytometer (BD Pharmingen). Data were analyzed using FlowJo software (Treestar, San Carlos, CA).

### Statistical Analysis

GraphPad, Inc. Prism software was used to perform log-rank Kaplan-Meier statistical analyses on skin graft survival curves. For longitudinal analysis of T cell expansion and differentiation, two-way ANOVA tests were performed, followed by Tukey post-test on significant results. For analysis of cytokine producing cells and KLRG-1 expression, one-way ANOVA tests were performed, followed by Tukey post-test on significant results.

## Results

### Anti-CD154 and DST interact to protect allogeneic grafts from rejection

**Figure 1:**
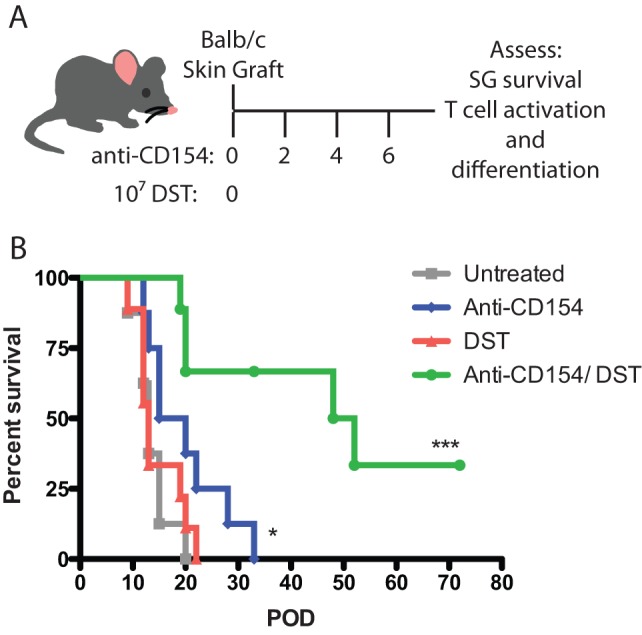
Anti-CD154 and DST interact to protect allogeneic grafts from rejection. A. B6-Ly5.2/Cr mice were transplanted with BALB/c skin grafts and were treated with 10^7^ BALB/c splenocytes (DST) and/ or anti-CD154 monoclonal antibody (500 μg on D0, 2, 4, 6), where indicated. B. Allo-skin grafts in untreated mice had an MST of 13 days. Monotherapy with either CD40/CD154 pathway blockade or DST led to rapid rejection of the allograft with MSTs of 17.5d (p = 0.039) and 13d (p = n.s.), respectively. Anti-CD154 and DST combined treated significantly prolonged allograft survival to 50 days (p = 0.0002). Data are summary of two experiments of 4–5 mice per group. *p<0.05, ***p<0.001.

Combined DST/MR-1 administration function in concert to prolong graft survival, but the independent contributions of these treatments to the innate and adaptive immune responses are still incompletely understood. Here, we examined the individual contributions of DST and anti-CD154 on innate and adaptive cell recruitment, the accumulation of inflammatory chemokines within the allograft, and the programming of alloreactive CD8^+^ T cell responses. This well-established regimen consists of BALB/c DST administered in conjunction with anti-CD154 monoclonal antibody treatment at the time of BALB/c skin transplantation onto naïve B6 recipients [6]. Using this protocol, untreated animals rapidly reject their skin grafts (MST 13d), while anti-CD154 mAb monotherapy results in a modest, but significant, delay in graft rejection as compared to untreated mice (MST 17d, p = 0.039), and DST monotherapy results in rapid allograft rejection, similar to untreated animals (MST 13d). In contrast, combined treatment with anti-CD154 mAb and DST results in significant prolongation of skin graft survival, as treated animals exhibited a MST of 50 days (p = 0.0002, compared to untreated mice) (Figure 1B).

### Anti-CD154 treatment alters recruitment of graft-infiltrating cells following transplantation

**Figure 2:**
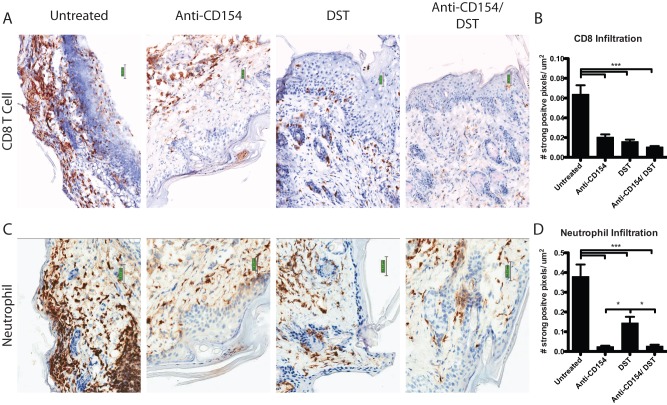
Anti-CD154 treatment alters recruitment of graft-infiltrating cells following transplantation. B6.SJL mice were transplanted with BALB/c skin grafts and were treated with 10^7^ BALB/c DST and/ or anti-CD154 mAb, where indicated. Day 7 explanted allo-skin grafts were stained for (A) CD8 and (C) Ly6b to determine the level of CD8^+^ T cell and neutrophil infiltration, respectively. Histological analyses of (B) CD8^+^ T cell and (D) neutrophil infiltration were digitally measured in 8–17 fields of epidermis and dermis. The ratio of total strongly positive pixels to total area was determined. Data are a summary of two experiments with three mice per group. Values are mean ± SEM. **p<0.01, ***p<0.001.

In order to assess the impact of DST and/or CD154 blockade on cellular infiltration into the graft, we measured the level of infiltrating CD8^+^ T cells and neutrophils in explanted allografts on day 7 post-transplantation via immunohistochemical staining for CD8^+^ T cells (Figure 2A) and neutrophils (Figure 2C). Quantification of the number of strongly positive pixels per μm^2^ revealed that either DST or CD154 blockade individually or in combination diminished CD8^+^ T cell infiltration into allografts (Figure 2B). In contrast, only anti-CD154 treatment resulted in profound diminution of neutrophil-specific anti-Ly6b staining in the transplanted allografts (Figure 2D).

### CD154 blockade decreases CXCL1, CCL3, and CCL5 expression in allografts

**Figure 3:**
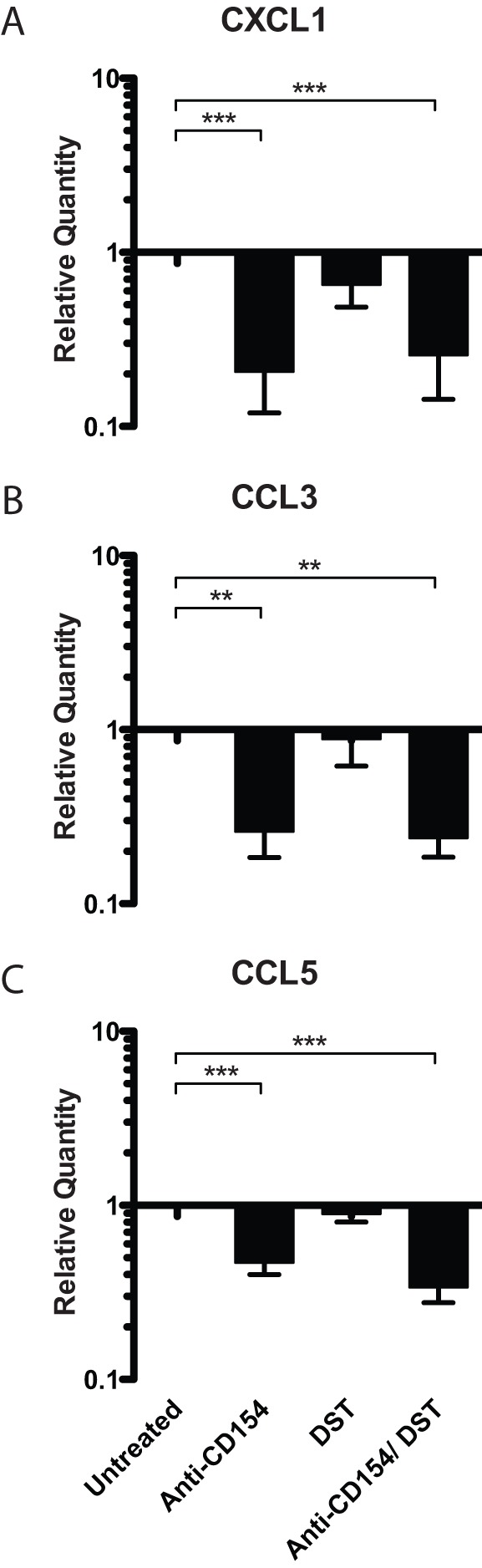
CD154 blockade decreases CXCL1, CCL3, and CCL5 expression in allografts. B6.SJL mice were transplanted with BALB/c skin grafts and were treated with 10^7^ BALB/c DST and/ or anti-CD154 mAb, where indicated. On day 7, skin grafts were explanted and processed for RNA extraction. Real time PCRs for chemokines CXCL1/ KC, CCL3/ MIP-1α, and CCL5/ RANTES were performed from cDNA. Data are summary of two experiments with three mice per group. Values are mean ± SEM. **p<0.01, ***p<0.0001.

Given the differential migration of neutrophils into the transplanted allografts in the presence of CD154 blockade vs. DST, we aimed to determine whether CD40/CD154 pathway blockade and DST differentially influenced the expression of chemokines in graft tissue. We interrogated the expression of CXCL1 (KC), CCL3 (MIP-1α), and CCL5 (RANTES) in allografts on day 7 post-transplantation by real-time PCR (Figure 3). The relative expression of KC/CXCL1, a primary neutrophil chemoattractant [19], was significantly attenuated in skin grafts of all animals treated with anti-CD154 mAb either as a monotherapy (0.21±0.09, p<0.0001) or in combination with DST (0.26±0.11, p<0.0001) when compared to untreated animals. In contrast, DST alone did not significantly reduce CXCL1 expression compared to untreated controls (Figure 3A).

Similarly, our results demonstrated that both MIP-1α and RANTES, molecules associated with the recruitment of both innate and adaptive immune cells including monocytes and T cells [19], were significantly attenuated in animals treated with CD154-blockade (Figure 3B, 3C). Again, DST did not statistically significantly diminish the expression of either MIP-1α or RANTES in the explanted grafts. Taken together, these results suggest that DST alone impairs adaptive CD8^+^ T cell responses but that only CD154 blockade attenuates expression of chemokines within the graft, thereby inhibiting neutrophil infiltration.

### Anti-CD154 and DST independently alter the expansion kinetics of activated CD44^high^ CD62L^low^ CD8^+^ T cells

**Figure 4:**
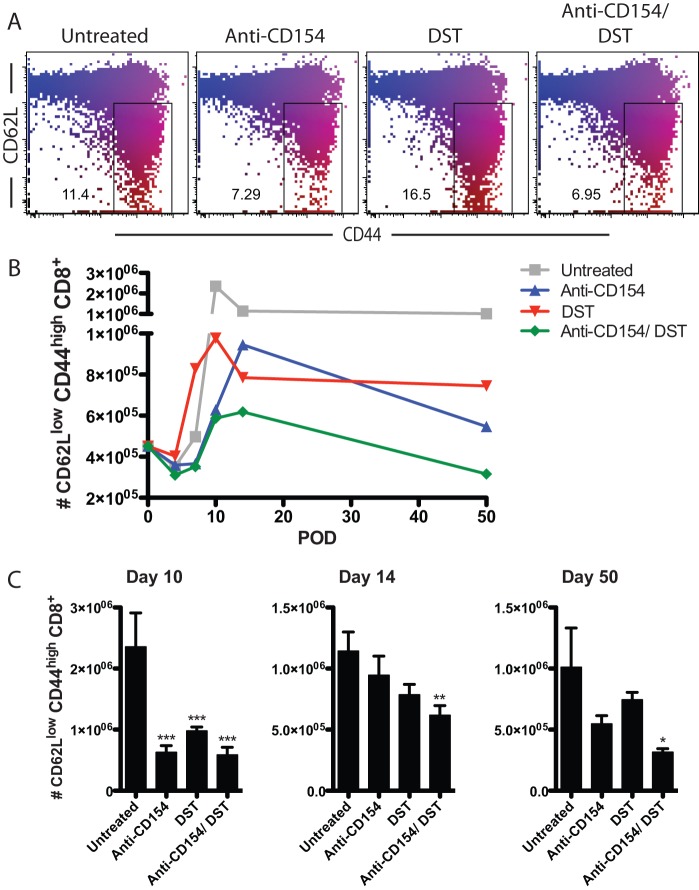
Anti-CD154 and DST independently alter the expansion kinetics of activated CD44^high^ CD62L^low^ CD8^+^ T cells. B6-Ly5.2/Cr mice were transplanted with BALB/c skin grafts and were treated with 10^7^ BALB/c DST and/ or anti-CD154 mAb, where indicated. A. Representative flow plots of CD44^high^ and CD62L^low^ CD8^+^ T cells isolated from spleens of mice on day 7 post-transplantation. B. Expansion kinetics of activated CD8^+^ T cells after allo-transplantation. C. Accumulation of CD44^high^ CD62L^low^ CD8^+^ T cells on day 10, 14, and 50 post-transplantation. Data are summary of two experiments with three mice per group. Values are mean ± SEM. *p<0.05, **p<0.01, ***p<0.001.

Next, we investigated the independent effects of DST and CD154 blockade on the magnitude and kinetics of the donor-reactive CD8^+^ T cell response following transplantation by tracking the absolute number of antigen-experienced CD44^high^ CD62L^low^ CD8^+^ T cells over time. In naïve B6 animals, approximately 0.45±0.07×10^6^ cells of the peripheral CD8^+^ T cell compartment were CD62L^low^ and CD44^high^ cells. Untreated recipients of allogeneic skin grafts developed large numbers of antigen-experienced cells with a peak of expansion at day 10 post-transplantation (2.36±0.55×10^6^) (representative flow cytometry data shown in Figure 4A). Following resolution of this effector cell population into memory, untreated recipients maintained 1.0±0.32×10^6^ effector/ memory phenotype T cells at day 50. Anti-CD154 mAb monotherapy resulted in a delayed expansion of activated CD8^+^ T cells, with a peak at day 14 (0.95±0.16×10^6^). Furthermore, the magnitude of this peak was significantly reduced compared with the peak response of untreated mice (day 10) (p = 0.0133). In contrast, DST monotherapy modestly accelerated the expansion of activated CD8^+^ T cells starting at day 7 (0.83±0.13×10^6^, p = 0.059), but also significantly reduced the peak expansion of activated CD8^+^ T cells compared to untreated controls (day 10: 0.98±0.07×10^6^, p<0.001). The combination of CD154 blockade and DST led to a significantly greater diminution of both the magnitude and kinetics of expansion of graft-reactive CD8^+^ T cells. In particular, the peak of expansion of these cells was delayed (day 14) compared with untreated controls. Furthermore, the magnitude of the peak of the alloreactive CD8^+^ T cell response following combined anti-CD154 and DST (day 14) was significantly diminished compared with the peak expansion of untreated mice (day 10) (0.62±0.08×10^6^ vs. 2.36±0.55×10^6^, respectively; p = 0.0026) (Figure 4B, C). In addition, compared with untreated animals, anti-CD154 and DST also significantly diminished the persistence of antigen-experienced CD44^high^ CD62L^low^ CD8^+^ T cells at memory time points (day 50) (1.01±0.32×10^6^ vs. 0.32±0.03×10^6^, respectively; p<0.05) (Figure 4B, C).

### Anti-CD154 and DST distinctly alter alloreactive CD8^+^ T cell programming into cytokine-producing effector cells

**Figure 5:**
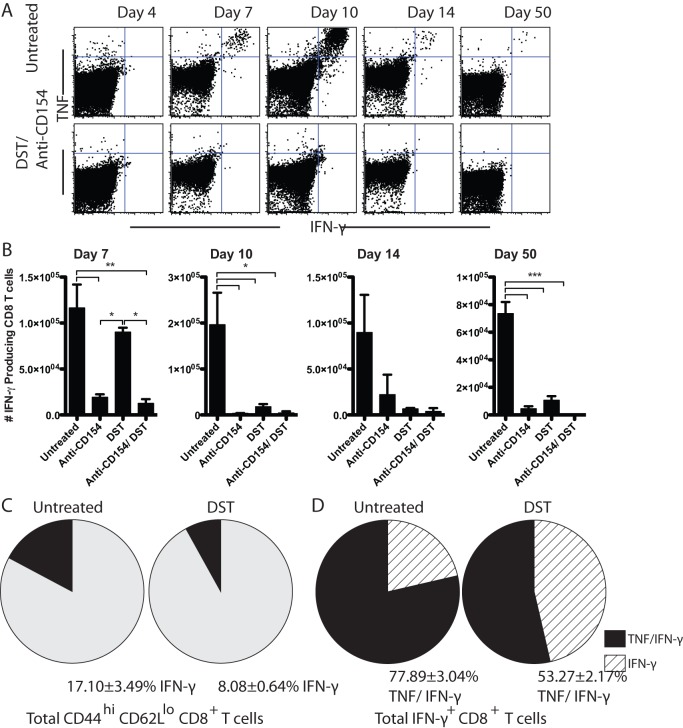
Anti-CD154 and DST distinctly alter alloreactive CD8^+^ T cell programming into cytokine-producing effector cells. B6-Ly5.2/Cr mice were transplanted with BALB/c skin grafts and were treated with 10^7^ BALB/c DST and/ or anti-CD154 mAb, where indicated. A. Representative flow plots of TNF and IFN-γ producing CD8^+^ T cells after *ex vivo* restimulation with BALB/c splenocytes, isolated from spleens of mice at day 7 post-transplantation. B. Absolute count of total IFN-γ producing CD8^+^ T cells in the spleen over time following *ex vivo* restimulation. C. Pie charts represent total activated CD44^high^ CD62L^low^ CD8^+^ T cells. The black wedges represent the frequency of activated CD44^high^ CD62L^low^ CD8^+^ T cells that produce IFN-γ on day 7 post-transplantation (p<0.05). D. Pie charts represent all IFN-γ producing CD8^+^ T cells. The striped wedges represent the IFN-γ-only producing population and black segments represent the TNF/IFN-γ double producing population in untreated vs. DST treated mice on day 7 (p = 0.0028). Data are summary of two experiments with three mice per group. Values are mean ± SEM. *p<0.05, **p<0.01, ***p<0.001.

To assess the effects of anti-CD154 and DST on the programmed differentiation of alloreactive CD8^+^ T cells into cytokine-producing effectors, splenocytes from skin grafted animals treated with anti-CD154, DST, or the combination were stimulated *ex vivo* with BALB/c splenocytes and subjected to intracellular cytokine staining (Figure 5). Data showed that as early as day 7 post-transplantation, splenic CD8^+^ T cells from untreated animals began to differentiate into multi-functional TNF and IFN-γ producing T cells. At day 10, untreated mice developed a peak of IFN-γ producing donor-reactive CD8^+^ T cells (1.96±0.70×10^5^) (Figure 5A, B). DST mediated an early expansion of IFN-γ producing graft-specific CD8^+^ T cells with a peak response at day 7 (0.90±0.05×10^5^) (Figure 5B). In contrast, anti-CD154 mAb monotherapy delayed CD8^+^ T cell differentiation into IFN-γ producing cells, such that a modest peak was observed 14 days post-transplantation (0.22±0.21×10^5^) (Figure 5B). Finally, combined treatment with anti-CD154 and DST significantly impaired the differentiation of antigen-specific CD8^+^ T cells into cytokine-producing donor-reactive T cells compared to untreated controls, such that IFN-γ^+^ alloreactive CD8^+^ T cells were virtually undetectable (Figure 5A, B).

We observed similar numbers of alloreactive IFN-γ-producing effectors in the untreated and DST treated groups on day 7 post-transplantation (Figure 5B). However, by day 10, this population expanded in the untreated animals, while it contracted in the DST treated animals. In order to understand the nature of the T cell programming that led to these disparate outcomes, we sought to determine the fraction of CD44^high^ CD62L^low^ activated T cells in these animals that produced cytokines following *ex vivo* restimulation with alloantigen. On day 7, 17.10±3.49% of antigen-experienced CD44^high^ CD62L^low^ CD8^+^ T cells isolated from untreated animals produced IFN-γ (Figure 5C, left). In contrast, although DST resulted in early accumulation of CD44^high^ CD62L^low^ CD8^+^ T cells, a significantly lower fraction of these cells had differentiated into IFN-γ producers on day 7 compared with untreated animals (8.08±0.64%, p<0.05) (Figure 5C, right).

These results indicated that DST decreased the frequency of differentiated IFN-γ producers as a percentage of the total activated T cell population. Therefore, we next interrogated whether this treatment also affected T cell differentiation into multi-cytokine producing effectors. To test this, IFN-γ producing CD8^+^ T cells were analyzed for their co-production of TNF. In untreated animals, the large majority of IFN-γ producing CD8^+^ T cells co-produced TNF (77.89±3.04%) upon restimulation. However, DST treatment significantly impaired the ability of graft-reactive CD8^+^ T cells to differentiate into IFN-γ^+^ TNF^+^ multi-cytokine producers compared with untreated controls (53.27±2.17%, p = 0.0028) (Figure 5D).

Similar to the effect on total burst size and differentiation of T cells, CD40/CD154 pathway blockade also impaired the development of alloreactive CD8^+^ memory T cells. Specifically, treatment with anti-CD154 resulted in a reduced accumulation of IFN-γ producing alloreactive CD8^+^ T cells at day 50 post-transplantation compared with untreated controls (0.45±0.17×10^4^ vs. 7.35±0.84×10^4^, respectively; p<0.0001). Similarly, DST monotherapy also diminished the persistence of IFN-γ producing alloreactive memory CD8^+^ T cells (1.07±0.03×10^4^, p<0.0001) at day 50 compared with untreated animals. Finally, the combination of CD154 blockade and DST reduced the alloreactive CD8^+^ memory T cell population more profoundly compared with untreated controls (0.02±0.02×10^4^ vs. 7.35±0.84×10^4^, respectively; p<0.0001) (Figure 5B).

### Anti-CD154 treatment increases the frequency of KLRG-1^high^ short-lived CD8^+^ effectors

**Figure 6:**
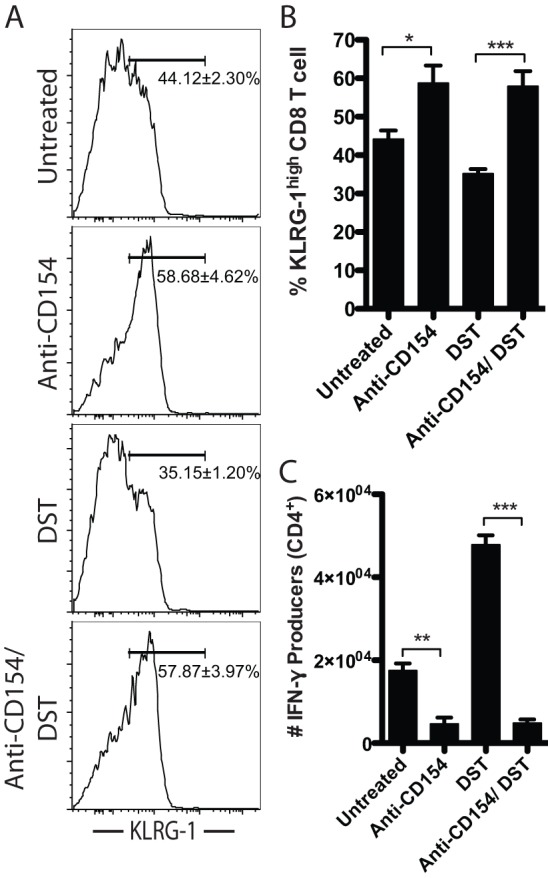
Anti-CD154 treatment increases the frequency of KLRG-1^high^ short-lived CD8^+^ effectors. B6-Ly5.2/Cr mice were transplanted with BALB/c skin grafts and were treated with 10^7^ BALB/c DST and/ or anti-CD154 mAb, where indicated. A. Flow plots of KLRG-1 expression on antigen experienced CD44^high^ CD62L^low^ CD8^+^ T cells at day 7 post-transplantation. B. Frequency of KLRG-1^high^ antigen experienced CD44^high^ CD62L^low^ CD8^+^ T cells on day 7. C. Absolute count of alloreactive CD4^+^ T cells producing IFN-γ on day 7. Data are summary of two experiments with three mice per group. Values are mean ± SEM. *p<0.05, **p<0.01, ***p<0.001.

These results indicate that CD154 blockade delays and diminishes the accumulation of alloreactive CD8^+^ T cells during the immune response to a transplant. In order to interrogate the molecular mechanisms underlying this effect, we examined the expression of a cell surface protein known to be associated with the differentiation of short-lived effector T cells that exhibit rapid contraction *in vivo*. The increased expression of KLRG-1 early during T cell responses has recently been shown to be upregulated on short-lived effector cells following antigen stimulation while the lower expression of KLRG-1 is associated with a long-lived memory T cell program [15]. We analyzed the expression of KLRG-1 on alloreactive CD8^+^ T cells on day 7 post-transplantation. In untreated animals, 44.12±2.30% of antigen-experienced CD44^high^ CD62L^low^ CD8^+^ T cells expressed increased levels of KLRG-1 by day 7. In contrast, CD154 blockade led to a marked reduction in the long-lived memory precursor KLRG-1^low^ CD8^+^ T cell population, and a commensurate increase in the frequency of KLRG-1^high^ short-lived effectors compared with untreated controls (KLRG-1^high^: 58.68±4.62% vs. 44.12±2.30%, respectively; p<0.05) (Figure 6A, B). DST monotherapy did not significantly alter the frequency of KLRG-1^high^ alloreactive CD8^+^ T cells (35.15±1.20%) (Figure 6A, B). Finally, the addition of anti-CD154 treatment to DST significantly increased the frequency of KLRG-1^high^ short-lived alloreactive CD8^+^ T cells compared with DST monotherapy (57.87±3.97% vs. 35.15±1.20%, respectively; p<0.001) (Figure 6A, B).

Lack of CD4^+^ T cell help has been associated with reduced memory CD8^+^ T cell development and survival following pathogen infection [20], [21]. Because we observed both an increase in KLRG-1^high^ CD8^+^ T cells early on and a diminution of alloreactive CD8^+^ T cells during memory timepoints, we hypothesized that anti-CD154-mediated inhibition of CD4^+^ T cell help may underlie these observations. We assessed the accumulation of alloreactive CD4^+^ T cells on day 7 post-transplantation by analysis of IFN-γ production following *ex vivo* restimulation. Results demonstrated that CD40/CD154 pathway blockade significantly reduced the accumulation of alloreactive IFN-γ^+^ CD4^+^ T cells compared with untreated controls (0.46±0.16×10^4^ vs. 1.75±0.18 10^4^, respectively; p<0.01) (Figure 6C). Furthermore, when anti-CD154 treatment was combined with DST, IFN-γ-producing CD4^+^ T cells were significantly reduced compared to DST monotherapy (0.49±0.09×10^4^ vs. 4.78±0.23×10^4^, respectively; p<0.001) (Figure 6C).

## Discussion

In this manuscript, we assessed the impact of CD154 blockade and DST on the programmed differentiation of alloreactive CD8^+^ T cell responses longitudinally following transplantation. We interrogated the effects of CD40/154 pathway blockade and DST treatment on adaptive and innate immune cell involvement in graft rejection. We demonstrated that treatment with anti-CD154 and DST induces distinct differentiation programs in alloreactive T cell populations; specifically that treatment with anti-CD154 not only delayed the expansion and accumulation of activated CD62L^low^ CD44^high^ CD8^+^ T cells, but also delayed and reduced CD8^+^ T cell differentiation into IFN-γ producing cells. Although the eventual emergence of alloreactive CD8^+^ T cells in anti-CD154 treated animals could be attributed to the waning effects of the antibody following cessation of treatment, this is not likely since *in vivo* administration of MR-1 has been shown to persist in animals with a half-life of 10.4 days [22]. Therefore, the delayed CD8^+^ T cell response in animals treated with CD40/CD154 blockade monotherapy is likely due to a CD154-independent breakthrough response, and not due to incomplete blockade of the pathway in this system.

Importantly, this study revealed a novel effect of CD40/CD154 pathway blockade on T cell differentiation, specifically the ability of CD154 blockade to increase the frequency of KLRG-1^high^ short-lived effector cells among CD8^+^ CD44^high^ CD62^low^ alloreactive effectors, and correspondingly decrease the frequency of KLRG-1^low^ long-lived memory precursors. KLRG-1 expressed early during an immune response is associated with a short-lived effector cell that is destined to die during the contraction phase of the response [15]. CD8^+^ T cell populations in CD154/DST treated animals contained fewer numbers of CD44^high^ CD62L^low^ activated effectors; therefore, these data suggest that one mechanism by which CD154 blockade might mediate deletion of this subset is through the induction of KLRG-1 expression. Previous studies have shown that increased expression of KLRG-1 can be attributed to increased antigen duration and increased inflammation [16], [23]. Further investigation into the mechanisms by which CD154 blockade also increases KLRG-1 expression in the context of transplantation is warranted.

In contrast, DST treatment significantly inhibited CD8^+^ T cell differentiation into competent IFN-γ secreting effectors, and of these IFN-γ-producing cells, a lower percentage of IFN-γ^+^ TNF^+^ double producers was observed compared with untreated controls. This pattern of cytokine expression is reminiscent of CD8^+^ T cell exhaustion, wherein T cells first lose the ability to make TNF, followed by the loss of IFN-γ, before being deleted altogether [24]. Because CD8^+^ T cell exhaustion is facilitated by exposure to high dose antigen, we posit that DST could be initiating a process similar to T cell exhaustion by exposing the cells to high dose antigen presented on relatively inert APCs [8].

Overall, our results suggest that the distinct effects of anti-CD154 and DST on T cell programming, namely to skew the antigen-specific population towards KLRG-1^hi^ short lived effectors and to induce a cytokine secretion pattern reminiscent of T cell exhaustion, respectively, functioned in concert to profoundly attenuate antigen-specific T cell responses. This was true both in terms of numbers of activated alloreactive CD8^+^ T cells and IFN-γ producers, at all time points over the course of the immune response to the allograft. Thus, our data suggest that the blunted T cell differentiation observed in DST treated recipients, when combined with the inhibitory signals associated with CD40/CD154 pathway blockade, produces a catastrophic exhaustive event for the cell, resulting in failure to mount and maintain an effective CD8^+^ T cell response followed by prolonged protection of the graft.

In addition, we investigated the effects of CD40/CD154 pathway blockade on neutrophil recruitment in the graft, as other studies have previously demonstrated that depletion of PMNs alleviates cellular infiltration and prevents cardiac allograft rejection [25], and observed that CD154 costimulation blockade resulted in reduced intra-graft infiltration of neutrophils. The ability of CD154 blockade to impair innate immunity has also been observed in other systems. For example, in a murine model of arterial vessel injury, treatment with anti-CD154 monoclonal antibodies significantly impaired innate immune cell infiltration into the carotid arteries [26]. In an antigen non-specific model of ischemia and reperfusion injury in liver transplants, Shen *et* *al.* demonstrated that animals treated with anti-CD154 had reduced injury to livers compared to untreated or anti-IFN-γ treated animals [27]. Taken together, our data demonstrate that not only are CD8^+^ T cell responses inhibited by CD154 blockade, but also innate immune cell trafficking into allografts may be dependent on CD40-CD154 interactions.

From these data, we conclude that anti-CD154 and DST work through distinct mechanisms to inhibit the expansion and differentiation of donor-reactive CD8^+^ T cells and recruitment of innate immune cells, resulting in prolonged graft survival following transplantation. While the use of intact FcR-binding anti-CD154 is not a clinically applicable approach due to the concerns for thromboembolism, blockade of this pathway remains a uniquely effective method of inhibiting graft rejection in experimental models. Current work to translate this approach to a clinically viable strategy has included the development of anti-CD40 monoclonal antibodies and RNAi-based approaches to inhibit CD40 expression [28], [29]. This RNAi approach could also be adapted for CD154 inhibition. Alternatively, non-cross-linking mAbs could be developed to antagonize CD154, similar to the development of nonactivating single chain F_V_-based reagents as a substitute for cross-linking anti-CD28 mAbs [30].

Thus, as renewed interest in therapeutic blockade of the CD154/CD40 pathway gains momentum due to promising results using anti-CD40 monoclonal antibodies in translational models [31], understanding the cellular and molecular mechanisms by which blockade of the CD154/CD40 pathway functions to inhibit alloreactive T cell responses is critical to guide rational development of immunosuppressive regimens incorporating these therapeutics for use in transplantation.
